# EGFR-Targeted Cellular Delivery of Therapeutic Nucleic Acids Mediated by Boron Clusters

**DOI:** 10.3390/ijms232314793

**Published:** 2022-11-26

**Authors:** Damian Kaniowski, Justyna Suwara, Katarzyna Ebenryter-Olbińska, Agata Jakóbik-Kolon, Barbara Nawrot

**Affiliations:** 1 Centre of Molecular and Macromolecular Studies, Polish Academy of Sciences, Sienkiewicza 112, 90-363 Lodz, Poland; 2 Department of Inorganic, Analytical Chemistry and Electrochemistry, Faculty of Chemistry, Silesian University of Technology, Krzywoustego 6, 44-100 Gliwice, Poland

**Keywords:** antisense oligonucleotide, ASO anti-EGFR, boron cluster, cancer cells, B-ASO, EGFR, uptake, drug delivery, BNCT, ICP MS

## Abstract

New boron carriers with high boron content and targeted cancer-cell delivery are considered the first choice for boron neutron capture therapy (BNCT) for cancer treatment. Previously, we have shown that composites of antisense oligonucleotide and boron clusters are functional nanoparticles for the downregulation of expression of epidermal growth factor receptor (EGFR) and can be loaded into EGFR-overexpressing cancer cells without a transfection factor. In this study, we hypothesize that free cellular uptake is mediated by binding and activation of the EGFR by boron clusters. Proteomic analysis of proteins pulled-down from various EGFR-overexpressing cancer cells using short oligonucleotide probes, conjugated to 1,2-dicarba-*closo*-dodecaborane (1,2-DCDDB, [C_2_B_10_H_12_]) and [(3,3′-Iron-1,2,1′,2′-dicarbollide)^−^] (FESAN, [Fe(C_2_B_9_H_11_)_2_]^−^), evidenced that boron cage binds to EGFR subdomains. Moreover, inductively coupled plasma mass spectrometry (ICP MS) and fluorescence microscopy analyses confirmed that FESANs-highly decorated B-ASOs were efficiently delivered and internalized by EGFR-overexpressing cells. Antisense reduction of EGFR in A431 and U87-MG cells resulted in decreased boron accumulation compared to control cells, indicating that cellular uptake of B-ASOs is related to EGFR-dependent internalization. The data obtained suggest that EGFR-mediated cellular uptake of B-ASO represents a novel strategy for cellular delivery of therapeutic nucleic acids (and possibly other medicines) conjugated to boron clusters.

## 1. Introduction

The epidermal growth factor receptor (EGFR), also known as HER1 or ErbB1, is a member of the tyrosine kinase receptor (RTK) family and exerts critical functions in maintaining physiological homeostasis [1]. Large, ligand-induced conformational changes of these transmembrane receptors lead to their homo- or hetero-dimerization, followed by cellular internalization. Subsequent activation of cytoplasmic catalytic activity leads to autophosphorylation of key tyrosine residues that serve as docking sites for several downstream intracellular effector proteins in the signaling cascade. Key signaling cascades activated after EGFR dimerization and tyrosine kinase pocket phosphorylation include pathways mediated by the Ras-RAF-MAP kinase pathway, phosphatidylinositol 3-kinase (PI3K), the serine/threonine kinase AKT, phospholipase C (PLC) and signal transducer, and activator of transcription 3 (STAT-3). Then these signaling pathways activate various biological outputs that are beneficial for different cellular processes such as angiogenesis, proliferation, differentiation, motility, survival and apoptosis [2,3]. Among all ligands that can bind and activate EGFR, three main entities can be distinguished: epidermal growth factor (EGF), heparin-binding EGF-like growth factor, and transforming growth factor (TGF-α) [4,5]. In healthy tissues, the availability of EGFR ligands is tightly regulated and the kinetics of cell survival, proliferation, and differentiation are precisely tailored to the tissue’s need to maintain homeostasis [6].

EGFR is not only essential for normal physiological activities, but is also involved as an oncogenic driver in the development and spread of various cancers [7]. In the tumor microenvironment, the EGF receptor is often constantly stimulated, either by the sustained production of EGFR ligands or by a mutation in EGFR itself that keeps the receptor in a state of constant activation [8]. Abnormal activation or expression of EGFR or its ligands promotes tumor growth, invasion, and metastasis, leading to a more aggressive phenotype and is often associated with poor prognosis and shorter survival [9,10,11]. Increased EGFR activity is characteristic of a number of tumors such as glioblastoma, non-small cell lung cancer (NSCL), head and neck squamous cell carcinoma (HNSCC), and breast, colon, ovarian, prostate, or pancreatic cancer [7,12,13]. Dysregulation of EGFR signaling during tumorigenesis is also responsible for drug resistance in cancer treatment. In particular, the EGFR variant III (EGFRvIII) with a deletion of exons 2–7 in glioblastoma, which is frequently co-expressed with the wild-type receptor (wtEGFR) in association with STAT-3, plays a key role in the resistance of cancer cells to chemotherapy and radiotherapy [14,15,16,17,18].

EGFR is considered an established target for cancer therapy. Many anti-EGFR treatment strategies have been approved or are in clinical progress. These include neutralizing monoclonal antibodies (mABs), small molecule tyrosine kinase inhibitors (TKIs), phosphoinositide 3-kinases (PI3K) inhibitors, and antisense nucleic acids for gene therapy [19]. Anti-EGFR monoclonal antibodies bind to the extracellular domain of inactive EGFR, thus blocking the ligand-binding region and ligand-induced activation of EGFR tyrosine kinase. These classes of anti-EGFR drugs currently widely used in cancer treatment include cetuximab (humanized IgG), panitumumab (human IgG2), and necitumumab (human IgG), Food and Drug Administration (FDA)-approved mABs [20,21]. The second class of anti-HER1 drugs, small molecule tyrosine kinase inhibitors (TKIs), are ATP-competitive molecules that bind to the intracellular catalytic domain of EGFR, thereby inhibiting EGFR autophosphorylation and altering downstream signaling pathways. TKIs are divided into first-generation reversible inhibitors targeting EGFR and HER2 (gefitinib, erlotinib, lapatinib), second-generation irreversible inhibitors (afatinib, dacomitinib, neratinib), and third-generation inhibitors, which are improved drugs to overcome resistance to first-generation TKIs in the treatment of NSCLC (AZD9291, CLO-1686) [22].

Another very promising strategy for cancer therapy via the EGFR downregulation is EGFR-directed gene therapy. The efficacy of oligonucleotide-based drugs such as antisense oligonucleotides (ASOs), G-rich antisense oligonucleotides (G4-ASOs), and small interfering RNAs (siRNAs) for executing the inhibition of protein synthesis has been widely demonstrated [23,24,25,26,27,28,29]. ASOs can be directed towards specific mRNA or pre-mRNA transcripts and in this way prevent translation either by steric blocking, splice-switching [30], or by mRNA cleavage using RNase H [31]. Exogenously introduced short interfering RNAs (siRNAs) perform RNA interference (RNAi) by incorporating their antisense strand into the RISC complex and degrading the target mRNA. The major challenge in fulfilling the potential of nucleic acid-based therapeutics is their intracellular instability and negative charge, which are the main obstacles to effective cell membrane penetration. To overcome problems during delivery, nucleic acids are often encapsulated in vehicles such as lipid nanoparticles (LNPs) and polymer complexes. At the cellular level, drugs must penetrate the extracellular matrix and pass through the cell membrane by receptor-mediated endocytosis. The encapsulated cargo must escape the endosome and avoid lysosomal degradation. Systemically administered nucleic acid drugs must be resistant to serum nucleases and degradation by macrophages within the reticuloendothelial system [32,33].

Targeted delivery has always been a major concern in the clinical use of therapeutic nucleic acids. Various monoclonal antibodies (mAbs) or mAb fragments against specific tumor antigens have been used as ligands for targeted delivery of siRNA to the tumor [34,35]. The EGF receptor has also been considered for targeted delivery to cancer cells. In normal cells, EGFR expression ranges from 4 × 10^4^ to 10^5^ receptors per cell [36], whereas epidermoid cell carcinoma lines (A431) express up to 2 × 10^6^ EGFR molecules per cell [37]. Because of these properties, EGFR has been widely used for EGFR-mediated delivery of various medical agents, as demonstrated for nanoformulated photosensitizers [38], anticancer drugs [39], polyplexes [40], and other compounds. For example, Kim and others have shown that coupling cetuximab, an antibody against EGFR, to the liposomal surface enhances delivery of siRNA to target mice with SK-OV-3 tumor xenografts. The developed anti-EGFR immunolipoplexes and immunoviroplexes showed remarkable cell binding and siRNA delivery to EGFR-expressing tumor cells compared with immunoliposomes and immunovirosomes. In particular, anti-EGFR immunoviroplexes exhibited the most efficient siRNA transfection into tumor cells, resulting in significant inhibition of tumor growth. Moreover, administration of doxorubicin in combination with anti-EGFR immunoviroplexes resulted in a remarkable and synergistic anticancer effect [41].

In the course of developing antisense oligonucleotides with novel scaffolds, we designed antisense oligonucleotides directed towards mRNA of EGFR decorated with boron clusters (B-ASOs) for boron neutron capture therapy (BNCT) [23,24,25]. Some of the tested molecules were found to be highly active inhibitors of *EGFR* gene expression, depending on the boron cluster charge (type of cluster), number of clusters, and site of oligonucleotide chain modification. Continuing this theme, novel self-assembling DNA nanostructures containing boron clusters and the same antisense oligonucleotide were developed [26,27]. These boron-based nanostructures were found to be active *EGFR* gene silencers and, more interestingly, they were able to penetrate A431 cells when used at a relatively high concentration but without transfection agents, as demonstrated by inductively coupled plasma mass spectrometry (ICP MS).

This puzzling observation motivated us to further investigate and confirm our hypothesis that the observed uptake in the absence of transfection agents is triggered by the boron clusters that could bind and activate the EGF receptor and thus mediate lipofectamine-free intracellular delivery. To this end, a series of boron cluster-containing probes were synthesized and used for pull-down experiments in protein lysates of various EGFR-overexpressing cancer cells to investigate the ability of boron clusters to bind to EGFR. In addition, a previously characterized antisense oligonucleotide with high metallacarboranes (FESANs) decoration was used to assess its lipofectamine-free cellular uptake in various normal and cancer cells, including those after EGFR silencing [24]. The assessment performed by ICP MS allowed us to determine the level of accumulated boron in the tested cells. The data obtained suggest that boron clusters bind to the EGFR and have the potential to deliver the conjugated cargo (oligonucleotides or possibly other molecules, including anticancer drugs) to EGFR-overexpressing cancer cells via the EGFR-targeted pathway.

## 2. Results

### 2.1. Chemistry

Two groups of compounds were synthesized to carry out the project (Table 1). Protein-specific pull-down probes were the first group, which consists of a short 2′-OMe oligonucleotides 5′-(GAU_B_UC)-3′ (1) conjugated to a boron cluster in the middle uridine unit (U_B_) and tagged with a biotin moiety at the 5′-end. The introduction of 2′-OMe chemical modifications was necessary to stabilize this construct against nucleolytic cleavage.

**Figure 1:**
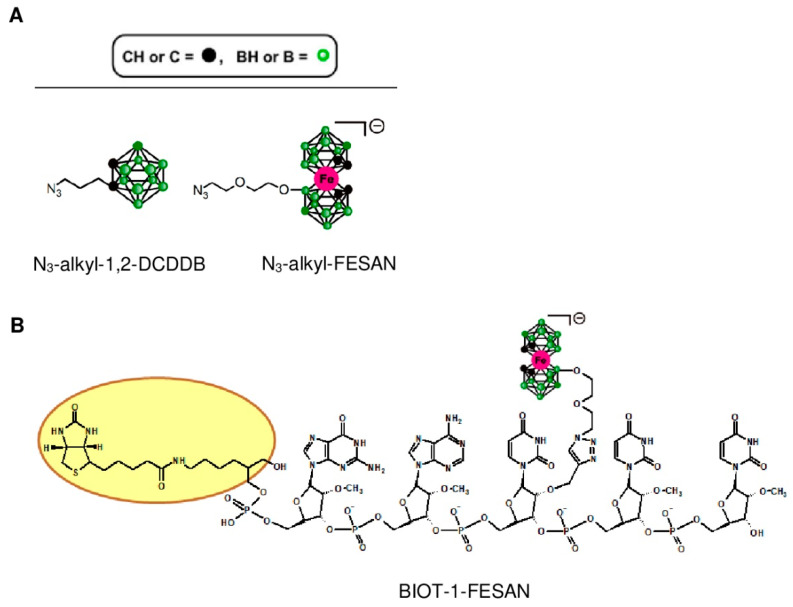
Structures of alkyl azide derivatives of boron clusters used in this studies are shown: N_3_-alkyl-1,2-dicarba-*closo*-dodecaborane (N_3_-alkyl-1,2-DCDDB, [C_2_B_10_H_12_]) and N_3_-alkyl-[(3,3′-Iron-1,2,1′,2′-dicarbollide)^−^]-(N_3_-alkyl-FESAN), [Fe(C_2_B_9_H_11_)_2_]^−^) (**A**). Scheme of the BIOT-1-FESAN fishing rod with biotin (yellow tag) and boron cluster (FESAN) bait. Illustrations prepared with the use of ADC/ChemSketch (https://www.acdlabs.com/, accessed on 7 December 2018) (**B**).

Probes were conjugated to azide derivatives of 1,2-dicarba-*closo*-dodecaborane (N_3_-alkyl-1,2-DCDDB, [C_2_B_10_H_12_]) or [(3,3′-Iron-1,2,1′,2′-dicarbollide)^−^] (N_3_-alkyl-FESAN), [Fe(C_2_B_9_H_11_)_2_]^−^ (Figure 1A), which were synthesized according to the procedures available in the literature [42,43]. Their structure and purity were confirmed by different spectral and chromatographic methods. 1,2-DCDDB is the organoboron compound that contains two carbon atoms in ortho-position, while metallacarborane consists of two 1,2-dicarba-*nido*-undecaborate residues ([C_2_B_9_H_11_]^2−^) that sandwich with iron (Fe^3+^) cations.

The second group of oligonucleotides were *EGFR* mRNA-targeted unmodified ASO (ASO anti-EGFR) and boron cluster-containing ASO (B-ASO), previously developed as inhibitors of gene expression in cellular systems [23,24] and their 5′-fluorescein-labelled version (FAM-B-ASO). In this B-ASO, five residues [(3,3′-Iron-1,2,1′,2′-dicarbollide)^−^] (FESAN) were used as the decorating moiety.

The synthesis of the oligonucleotides modified with boron clusters was performed in a two-step procedure, in line with the general approach developed previously [23] in which routinely obtained 2′-*O*-propargyluridine (U_Pr_)-containing oligodeoxyribonucleotides were post-synthetically conjugated with an alkyl azide derivative of the boron cluster using a copper-catalysed Huisgen-Meldal-Sharpless azide-alkyne cycloaddition (also known as a click reaction) [43,44,45,46].

The final products were purified by reverse phase-high performance liquid chromatography (RP-HPLC, see Figures S1–S4) and their molecular mass and purity were confirmed by matrix-assisted laser desorption/ionization time-of-flight mass spectrometry (MALDI TOF MS).

### 2.2. Pull-Down Assay to Identify the Pool of Proteins Interacting with the Boron Cluster Bait and Proteomic Analysis by Liquid Chromatography-Mass Spectrometry LC-MS

**Figure 2:**
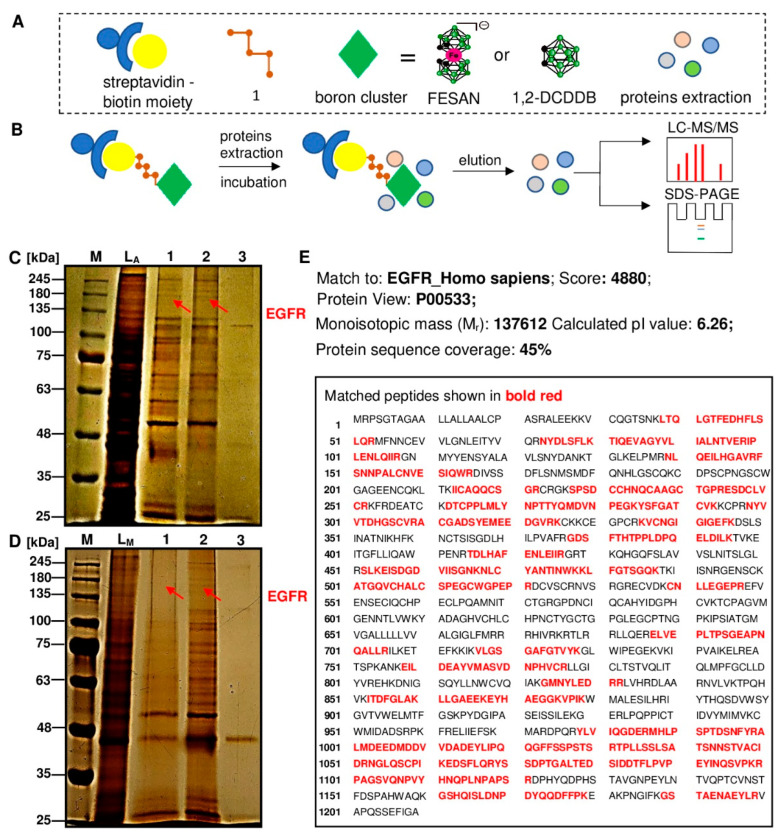
Schematic representation of the model ASO with fishing rod (BIOT-1) with boron cluster bait (U_B1_ (FESAN) or U_B2_ (1,2-DCDDB)) which was used to isolate cellular proteins with specific affinity (**A**,**B**). SDS-PAGE analysis of proteins eluted with BIOT-1-FESAN (**C**,**D**; lane 1), BIOT-1-1,2-DCDDB (lane 2), and BIOT-1 (control) (lane 3). Total protein mixture of epidermoid squamous cell carcinoma A431 (**C**; lane L_A_) and mouse embryonic fibroblast MEF-WT cells (**D**; lane L_M_). Size marker, silver staining of eluted proteins resolved to SDS-PAGE (**C**,**D**; lane M). Proteomic analysis of EGFR-band protein sequence by LC-MS/MS analysis. Identified amino-acid chains (bold red) match EGFR_Homo Sapiens from Mascot (version 1.9) proteomic analysis (**E**).

The aim of this experiment was to isolate the pool of proteins that interact with the specific boron clusters and to see if we could identify among the proteins with a molecular weight of about 180 kDa (corresponding to the M.W. of EGFR) the candidate protein that could be an EGF receptor. To achieve this goal, we developed a simple probe called “BIOT-1 fishing rod with boron cluster bait” as described above (Table 1 and Figure 1B). In this experiment, we used a total cell protein lysate from two cell lines: EGFR-overexpressing human epidermoid carcinoma cells (A431) and normal cells from mouse embryonic fibroblasts (MEF-WT). BIOT-1 probes (with U_B.1_ or U_B.2_ uridine-boron clusters) and BIOT-1 as a control fishing rods without boron clusters were immobilized on streptavidin agarose beads and used in pull-down experiments (Figure 2B). After 2 h of incubation with gentle mixing, the beads were separated from the cell protein lysates, washed, and heat denatured to release the bound proteins. The fraction of isolated proteins was analysed by sodium dodecyl sulphate-polyacrylamide gel electrophoresis (SDS-PAGE) (4% stacking gel, 8% resolving gel).

The scan of the gel of the isolated proteins from each pull-down experiment is shown in Figure 2C (A431 cells) and Figure 2D (MEF-WT cells). In these gels lanes, L_A_ and L_M_ correspond to the total protein mixture from A431 or MEF-WT cells, respectively. Lanes 3 (Figure 2C,D) show the pool of proteins isolated from the affinity experiment using the BIOT-1 as a control bait. Lanes 1 and 2 represent protein mixtures isolated from the cells with BIOT-1-FESAN (lines 1) and BIOT-1-1,2-DCDDB (lines 2). Both tested probes show high selectivity towards similar proteins with different molecular weights (MWs), among them the most representative: 25–30, 40, 55, 70, 100, 180, and 245 kDa. The nonspecific affinity product bound to the BIOT-1 control probe (without boron cluster) was also present in pools specific for all boron cluster-containing probes (Figure 2C,D, lane 3). In general, however, we found that the pools of proteins binding to boron clusters were very similar regardless of the cluster tested and contained a very specific group of proteins.

The next step of the current study was proteomic analysis and confirmation of the protein type of EGFR bands (180 kDa) from SDS-PAGE (Figure 2C,D, red arrows) for the two types of boron cluster fishing rod: BIOT-1-FESAN and BIOT-1-1,2-DCDDB. The proteomic profile of the EGFR band sample was generated using the LC-MS analysis platform (Figure 2E). Briefly, bands potentially specific for EGFR proteins were extracted from each sample and then digested with trypsin to obtain shorter peptides of which amino acid sequences were analysed by LC-MS mass spectrometry. Proteomic analysis of the amino acids sequences was performed using the raw files of the samples from a Mascot version 1.9 database search engine (Matrix Sciences, London, UK) and matched with identified proteins in the Swiss-Prot protein database. The obtained results confirmed that the bands of protein isolated from SDS-PAGE correspond to EGF receptor (UniProtKB-P00533 (EGFR_HUMAN)) with a chain length of 1210 amino acids and a protein sequence coverage of 45% (Figure 2E).

### 2.3. Cellular Accumulation of Boron Is Modulated by EGFR Overexpressed in Cancer Cells

Selective cellular uptake plays an important role in the delivery of anticancer drugs to the target cells. Internalization of boron clusters via EGFR-targeted receptors, resulting in boron accumulation in cancer cells overexpressing this receptor, may be a promising approach to treat tumors using BNCT [47]. Recent reports suggest that as little as 5–20 µg of ^10^B/g cells in human melanoma cells is sufficient to produce a therapeutic effect in BNCT [48]. This amount of boron ^10^B isotope can be lower if the boron delivery system is concentrated in or near the nucleus. For BNCT to be successful, the boron agent must meet the most important conditions, namely selective uptake by tumor tissue compared to normal tissue (preferably accumulation in the specific substructure of tumor cells) with ideal tumor-to-normal tissue and tumor-to-blood ratios of 2.5:1 and 5:1, respectively. Another important factor is a rapid excretion of boron compounds from blood and healthy tissue [49]. For this reason, we evaluated the ability of carrier-free uptake of antisense oligonucleotide conjugated with 5 metallacarboranes (FESANs) residues (B-ASO) directed toward mRNA of EGFR [24] by inductively coupled plasma mass spectrometry analysis (ICP MS). The tests were performed on cancer cells with different levels of expression of EGFR (A431, U87-MG, HTC-116, HeLa, MG-63, MCF-7) and normal cells (HEK-293, CCD-841-CoN, MEF-WT, h-FOB 1.19, NHDF) as well as on human macrophages (three independent donors).

Because ASOs lack a chemical modification that increases nuclease stability, B-ASO was tested in an in vitro experiment using basic cell culture media (without FBS) at 37 °C. The results for the stability of B-ASO in these conditions from 0 to 48 h, performed by 20% denaturing PAGE analysis, are available in the supplemental information (Figure S5).

**Figure 3:**
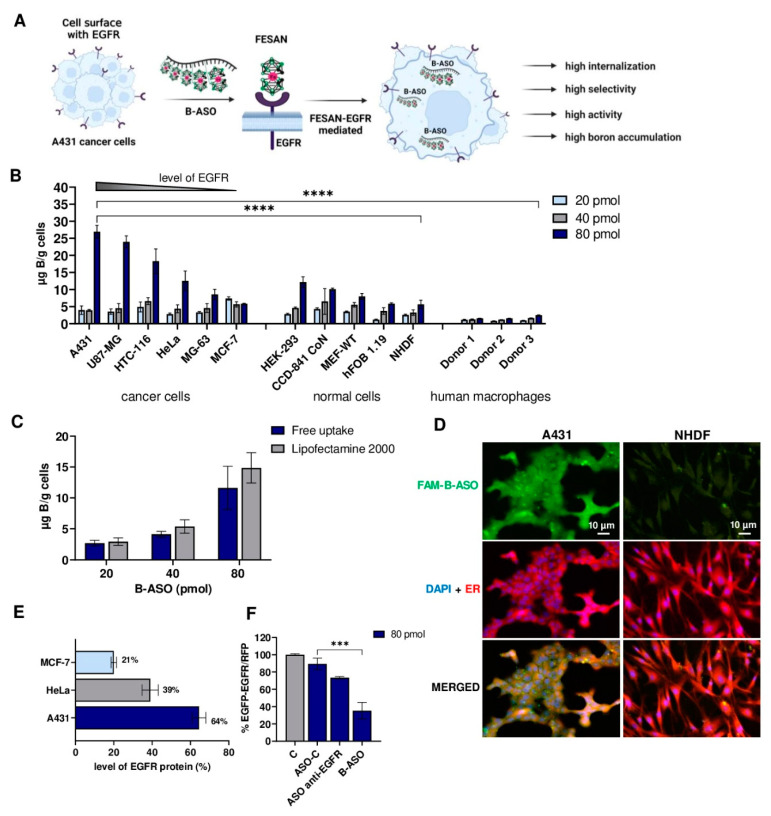
Representative illustration of the free uptake of B-ASO by EGFR-overexpressing cancer cells (**A**). Cellular uptake of B-ASO by different cancerous and normal cells as well as by primary human macrophages from monocytes derived from three independent donors measured using ICP MS; the means ± SEM (n = 3) are shown; **** *p* ≤ 0.0001 (**B**). Comparison of transfection efficiency of B-ASO with Lipofectamine 2000 or by free uptake (i.e., no transfection agent); the means ± SEM (n = 3) are shown (**C**). Representative fluorescence microscopy images of A431 epidermoid carcinoma skin cells (left images) and NHDF human dermal foreskin fibroblasts (right images) after incubation with FAM-B-ASO (80 pmol/well) at 37 °C for 12 h. Visualization of green fluorescent FAM-B-ASO is shown in row 1. Blue cell nuclei (DAPI) merged with red ER membranes (ER-Tracker Red) and green fluorescent FAM-B-ASO are shown in rows 2 and 3, respectively (n = 3). All panels were magnified 20× (**D**). Analysis of EGFR protein level expression relative to the actin expression level (100%) in A431, HeLa, and MCF-7 [51,52] cancer cells and quantification using immunoblot (Figure S9), which was measured using G-BOX apparatus; the means ± SEM (n = 2) are shown (**E**). Silencing activities of B-ASO, ASO anti-EGFR, and ASO-C (non-active ASO) (80 pmol/well) determined using a pEGFP-EGFR/RFP dual fluorescence assay in A431 cells. The cells were transfected with the pEGFP-EGFR and pDsRED-N1 plasmids (in the presence of Lipofectamine 2000) and then treated with the respective oligonucleotides (free uptake). The cells were then incubated for 48 h. The relative EGFP-EGFR/RFP fluorescence of the cells transfected with the plasmids only was assessed as 100% (C-Control); the means ± SEM (n = 3) are shown; *** *p* ≤ 0.001 (**F**). Figures were created in BioRender (https://biorender.com/, accessed on 23 June 2022) and GraphPad Prism (https://www.graphpad.com/, accessed on 15 August 2022).

In the current study, we investigated whether B-ASO containing five iron-sandwich boron clusters (one molecule of B-ASO contains 90 boron atoms) can be internalized together with EGFR and enter the cells via EGFR-dependent endocytosis (Figure 3A). For this purpose, we added B-ASO at different concentrations (20, 40, 80 pmol/well) to the culture of various cancer and normal cells (15 000/well) and then incubated at 37 °C for 12 h. Next, the cells were lysed and the boron content was determined by ICP MS. The calibration curves for the two stable boron isotopes (^10^B and ^11^B) were used to determine the content of boron delivered to the cells. The accumulation of boron in the cells may suggest an active uptake of B-ASO directly from the culture medium into the cells.

As shown in Figure 3B, B-ASO (80 pmol/well) was efficiently taken up from the medium by various cancer cells overexpressing EGFR compared to the normal cells or human macrophages where the uptake was lower since the natural expression level of EGFR is low. The highest concentration of boron was found in A431, reaching 27 µg B/g (5.4 µg ^10^B/g) cells. Glioblastoma cancer cells (U87-MG) took up 24 µg B/g (4.8 µg ^10^B/g). The lowest concentration of boron (6 µg B/g cells) was found in breast cancer cells (MCF-7). Interestingly, cancer cells exhibited a 2-fold greater concentration of boron compared to normal cells. The boron uptake concentration in human embryonic kidney cells (HEK-293) and human colon epithelial cells (CCD-841 CoN) was 13 µg B/g cells and 10 µg B/g cells, respectively. Low boron concentration in normal cells was also found in NHDFs (6 µg B/g cells) and human osteoblasts (h-Fob 1.19, 5 µg B/g cells). Interestingly, human macrophages derived from primary monocytes from three independent donors took up minimal B-ASOs reaching about 3 µg B/g cells.

Moreover, in this study, the distribution of boron reported as the ratio of boron in cancer cells to normal cells (C/N) and cancer cells to human macrophage cells (C/M) was carefully analyzed. The ratio of boron content in cancer cells to normal NHDF cells for A431, U87-MG, and HTC-116 cells was ~4.8, 4.3, and 3.2, respectively. In addition, the boron concentration ratio of the same cancer cells to three human macrophage cells was in the range of 11.8–17.6 (Figure S7).

Next, the delivery efficiency of B-ASO to HeLa cells by EGFR-targeted free uptake and commercial transfection agent (Lipofectamine 2000) were compared (Figure 3C). Of note, Lipofectamine 2000 cannot be used in human therapy [50]. The transfection efficiency of HeLa cells carried out with commonly used Lipofectamine 2000 (in the ratio of 2 µL/1 µg ASO) was similar to the free uptake of B-ASO mediated by EGFR (80 pmol).

To confirm that free uptake of B-ASOs is dependent on the level of EGFR expression, we prepared a fluorescent derivative of B-ASO, FAM-B-ASO (described in Material and Methods 1 and 2, chromatographic and spectral analysis is given in Figure S7), to assess the uptake and cellular localization of B-ASO. In this experiment, free uptake of FAM-B-ASO was monitored in A431 cancer cells exhibiting high EGFR expression levels compared to NHDFs with low EGFR density. Cells were treated with FAM-B-ASO (80 pmol/well) and after 12 h of incubation, cells were visualized by green fluorescence microscopy (Figure 3D). In parallel, the nuclei of the tested cells were stained with DAPI and the endoplasmic reticulum membranes were stained with ER-Tracker. A strong green fluorescent signal was observed in A431 cells, in contrast to normal NHDFs, which exhibited minimal or any fluorescence (Figure 3D, third panel from top). Therefore, we could conclude that FAM-B-ASO uptake is dependent on EGFR expression level and cell type. Additional results of FAM-B-ASO uptake by cancerous (U87-MG and HCT-116) and normal (MEF-WT and CCD-841) cells analyzed by fluorescence microscopy are provided in the supplemental information (Figure S9). These results confirm high levels of FAM-B-ASO uptake by U87-MG and HCT-116 cancer cells and low transfection efficiency of normal cells.

EGFR overexpression is a well-known characteristic feature of some cancers. The membrane microenvironment of EGFRs may play an important role in drug-receptor interactions. Quantification of EGFR expression shows that A431, HNSCC, HTC-116 cancers have the highest EGFR levels, whereas HeLa or MCF-7 cells possess lower expression [51,52]. EGFR protein expression in three cell lines - A431, HeLa, and MCF-7, was assessed by SDS-PAGE of the protein lysates and EGFR-specific immunoblotting (Figure S9). Additionally, the EGFR:ACTIN ratio was analysed quantitatively using immunoblot imaging (Figure 3E). EGFR content was highest in human epidermoid cell carcinomas (A431) compared to human cervical cancers (HeLa) or human breast cancers (MCF-7), as quantified by the ratio of EGFR-band vs actin-band intensities (%). The highest observed accumulation of boron correlated with the highest concentration of EGFR observed in A431 cells (Figure 3B,E).

Finally, the anti-EGFR antisense activity of the tested B-ASOs (80 pmol/well) was evaluated using the dual fluorescence assay (DFA) (Figure 3F) (described and used in our previous works [23,24,26,28]). B-ASOs were added to A431 cells without a commercial transfection agent. After 48 h of incubation, B-ASO was freely taken up by A431 cells via interactions between FESAN and surface receptors of the EGFR pathway. In doing so, the amount of exogenous EGFR-GFP protein was reduced by ~70% compared to the control ASO-C (non-active ASO) (Figure 3F). ASO anti-EGFR (without FESAN) was also taken up by A431 cells but in a negligible quantity and produced only a slight EGFR inhibitory effect. These results demonstrated that B-ASO is efficiently taken up by EGFR-overexpressing cancer cells, providing adequate boron concentrations for BNCT and inhibiting mRNA expression of the *EGFR* gene.

### 2.4. Reduction of EGFR Level in Cancer Cells Decreases the Free Uptake of B-ASO

**Figure 4:**
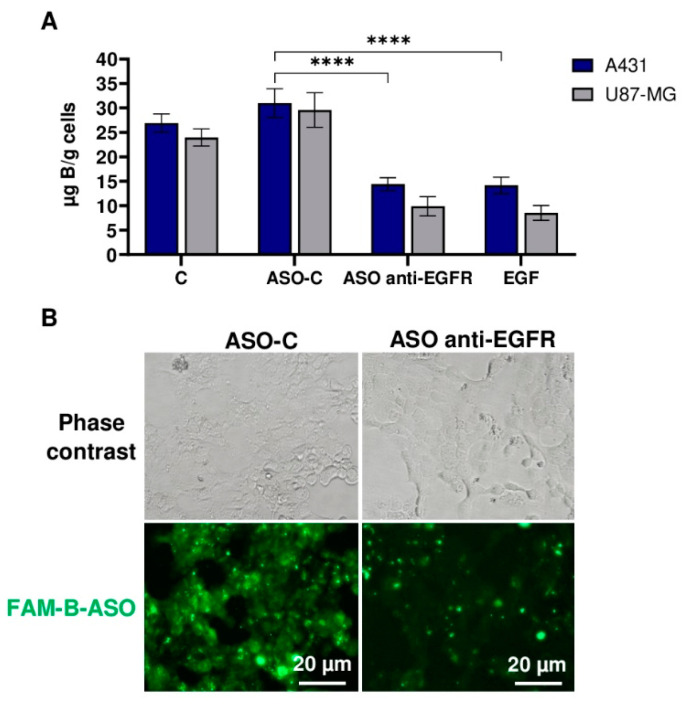
Boron-accumulation in A431 and U87-MG cells as a result of free uptake of B-ASO. In the first assay, the cells were transfected with ASO-C (non-active ASO) or ASO anti-EGFR (200 nM, respectively) in the presence of Lipofectamine 2000 [27]. After 36 h of incubation, the medium was replaced and cells were treated with B-ASO (80 pmol/well, free uptake). In the second assay free EGF ligand (10 ng/mL, 1 h incubation) was added to A431 and U87-MG cells to block B-ASO binding to EGFR. Then, the medium of the mixture was replaced with fresh medium and B-ASO (80 pmol/well, free uptake) was added. In both experiments, the cells were then incubated at 37 °C for 12 h and the concentration of boron in the cell lysates was measured using ICP MS. The means ± SEM (n = 3) are shown; **** *p* ≤ 0.0001. Figure was created in GraphPad Prism (https://www.graphpad.com/, accessed on 15 August 2022) (**A**). Representative fluorescence microscopy images of A431 cells transfected with ASO-C and ASO anti-EGFR (phase contrast) and FAM-B-ASO as described in panel A. Visualization of the green fluorescent FAM-B-ASOs was analyzed using fluorescence microscopy; (n = 3, bottom). All panels were magnified 20× (**B**).

To further confirm our hypothesis that EGFR mediates free cellular uptake of B-ASO through the receptor mechanism, we performed two additional experiments. First, EGFR expression was downregulated using antisense silencing of *EGFR* mRNA with ASO anti-EGFR. In the second experiment, EGFR was blocked with free EGF ligand. The concentration of the accumulated boron in the downregulated and blocked EGFR experiments was measured using ICP MS. The data obtained are summarized in Figure 4A.

In the experiments with free uptake of B-ASOs and with ASO anti-EGFR, expected to decrease endogenous *EGFR* mRNA levels [23,24], lower boron accumulation (16 and 10 µg B/g cells) was observed in A431 and U87-MG cancer cells than in the experiments without antisense EGFR silencing, where boron uptake was 27 and 24 µg B/g cells, respectively. In parallel, cancer cells were transfected with non-active ASO-C [24,27,28] and Lipofectamine 2000 and then treated with free B-ASO (80 pmol/well). A slight increase in boron content in the cancer cells was observed using ASO-C and Lipofectamine 2000 compared to the control experiment (without the use of oligonucleotides coated with a commercial transfection agent) (Figure 4A). Incubation of cancer cells with the free ligand EGF also reduced B-ASO binding (by competition) with the extracellular domain of EGFR, reaching boron cellular uptake levels of 14 and 8 µg B/g for A431 and U87 cell lines, respectively (Figure 4A). Therefore, blocking of EGFR in tumor cell lines (A431 and U87-MG) reduces the free uptake of B-ASO. Microscopic analysis further confirmed this result. Moreover, A431 cells transfected with ASO anti-EGFR exhibited lower uptake of FAM-B-ASO compared to cells transfected with ASO-C (Figure 4B).

As expected, EGFR increased the free uptake of B-ASO which is characteristic for cancer cells overexpressing EGFR.

### 2.5. Time Course of the Free Uptake of Boron Cluster Decorated FAM-B-ASO or FAM-1-FESAN

**Figure 5:**
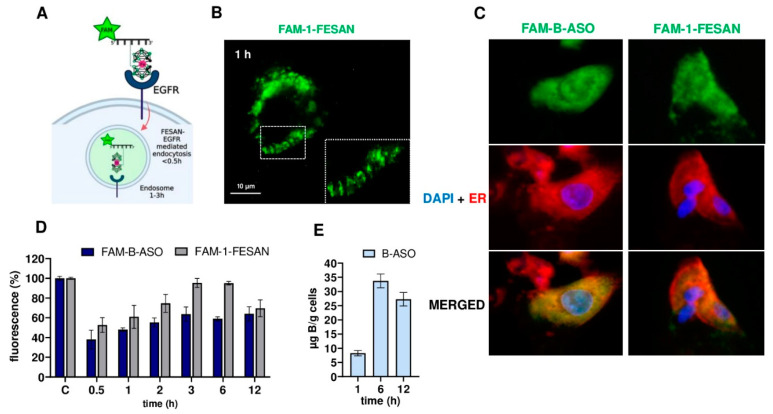
Design concept based on free uptake of FAM-1-FESAN mediated by FESAN-EGFR in A431 cells (**A**). The compound FAM-1-FESAN (80 pmol/well; 1 h incubation) is freely taken up by A431 cancer cells in vitro, as demonstrated using fluorescence microscopy; (n = 3) (**B**). Evaluation FAM-B-ASO and FAM-1-FESAN (80 pmol/well, 12 h incubation) localization in A431 cells using fluorescence microscopy. Visualization of green FAM-B-ASO (row 1) or FAM-1-FESAN (row 2) compounds, blue nuclei (DAPI), and red ER membranes (ER-Tracker Red) are shown. Merged fluorescence images of the FAM-B-ASO or FAM-1-FESAN, nuclei, and ER are shown in row 3; (n = 3). All panels were magnified 60× (**C**). The time course experiment measured the free uptake of FAM-B-ASO and FAM-1-FESAN (80 pmol/well; incubation time: 0.5, 1, 3, 6, 12 h) using a fluorescence microplate reader. FAM-B-ASO and FAM-1-FESAN (80 pmol/well) and itself without cells are a (C- control). The means ± SEM (n = 3) are shown (**D**). Boron concentration in lysates of A431 cells measured by ICP MS during the time course experiment after 1, 6, and 12 h of incubation with B-ASOs (80 pmol/well). The means ± SEM (n = 3) are shown (**E**). Figures were created in BioRender (https://biorender.com/, accessed on 23 June 2022) and GraphPad Prism (https://www.Graphpad.com/, accessed on 15 August 2022).

Understanding the interactions of boron compounds with A431 epidermoid carcinoma cells overexpressing EGFR (Figure 5A) provides an opportunity to improve the performance of B-ASO delivery without transfection reagents in the treatment of cancer using BNCT. Therefore, a time course study of free uptake of the conjugate FAM-1-FESAN (80 pmol/well) in A431 cells was observed using fluorescence microscopy after 0, 0.5, 1, 3, 6, and 12 h of incubation (Figure 5B and Figure S8). At the selected time points, cells were washed with buffered PBS and imaged using fluorescence microscopy. The FAM-1-FESAN compound showed very strong and efficient uptake over time. Within the first 0.5 h, cells showed a significant association with FAM-1-FESAN, mainly with the cell surface, and the amount of internalization increased with time. After 1 h of incubation, numerous green punctate structures appeared along the cell membrane, indicating that FAM-1-FESAN was trapped in endosomes (Figure 5B). These punctate structures were visible for up to 3 h after incubation (Figure S8). This result may indicate the free entry of FAM-1-FESAN by endocytosis via interaction with the EGFR. As time progressed, the total amount of fluorescent vesicles decreased, and FAM-1-FESAN was detected throughout the cytosol of the cells.

Using fluorescence microscopy, we examined the localization of FAM-B-ASO (five FESAN residues in the ASO chain) and FAM-1-FESAN (80 pmol/well) after 12 h of incubation in A431 cells (Figure 5C). In parallel, the nuclei of the tested cells were stained with DAPI and the endoplasmic reticulum membranes (ER) were stained with ER-Tracker Red. Careful analysis of the localization of the green boron compounds (FAM-B-ASO and FAM-1-FESAN) with blue nuclei and with red endoplasmic reticulum (merged panels) demonstrated that the fluorescent boron compounds are located in the cytoplasm of the cells where the B-ASOs exert their mRNA *EGFR* silencing activity [24].

Subsequently, a fluorescence microplate reader was used to quantitatively evaluate the uptake rate of FAM-B-ASO and FAM-1-FESAN (80 pmol/well) in A431 cells over time (0, 0.5, 1, 2, 3, 6, 12 h) in a basic culture medium (Figure 5D). The highest uptake of boron compounds (FAM-1-FESAN) was observed after 3 h (fluorescence level of 95%). After 3 h of FAM-1-FESAN incubation, the fluorescence began decreasing and reached 67% after 12 h of incubation. Interestingly, for FAM-B-ASOs, fluorescence increased rapidly. FAM-B-ASO fluorescence reached 66% after 3 h of incubation and increased to 70% after 12 h of incubation when freely taken up by A431 cells. ICP MS analysis confirmed greater free uptake of boron (B-ASO, 80 pm/well) in A431 cell lysates after 6 h of incubation (32 µg B/g cell) compared to 12 h of incubation (27 µg B/g cell) (Figure 5E). This ICP MS result is consistent with the fluorescence measurements for FAM-B-ASO and FAM-1-FESAN and indicates efficient accumulation of boron in A431 cells over time.

## 3. Discussion

In the approach described here, we have designed molecules based on a short fragment of a DNA oligonucleotide conjugated to boron clusters and a biotin moiety to identify proteins to which these molecules have high binding affinity. The biotin–streptavidin affinity system allowed the construction of a fishing rod with a boron cluster-bait, which could then be used to pull-down the boron-cluster entrapped proteins from a cell protein mixture. Human epidermoid carcinoma A431 and mouse embryonic fibroblast MEF-WT cell lines were selected for these experiments. Two types of boron clusters: 1,2-dicarba-*closo*-dodecaborane (1,2-DCDDB, C_2_B_10_H_12_) and 3,3′-Iron-bis (1,2-dicarbollide) (FESAN, [Fe(C_2_B_9_H_11_)_2_]^-^), were used as baits, and the DNA fragment of the rod was supplied with 2′-OMe units to protect the oligonucleotide from cellular exo- and endonucleases.

Using SDS-PAGE analysis and silver staining, the bands of proteins (Figure 2C,D) specifically bound to the boron cluster-baits BIOT-1-FESAN or BIOT-1-1,2-DCDDB (Table 1) in lysates of cancer and normal cells were identified and compared to bands obtained during whole protein extraction. In these studies, we focused on the 180 kDa protein band. The bait fishing rods bound (among others) to the 180 kDa protein from complete protein lysates of both the EGFR-overexpressed A431 cell line and normal MEF-WT cells (Figure 2C,D, lines 1 and 2). Therefore, we further investigated whether this ~180 kDa protein band corresponded to EGFR using proteomic LC-MS/MS analysis. As expected, the obtained LC-MS/MS results confirmed that the amino acid sequences of protein in this band match the EGFR protein (EGFR_Homo sapiens, Protein View: P00533) sequence. Moreover, the amino acid chain coverage was as high as 45%.

Previously, we confirmed the high affinity of the negatively charged FESAN residues, encompassing the entire ASO molecule, for crude snake venom proteins using microscale thermophoresis (MST) measurements [25]. Furthermore, we have shown that a single FESAN unit at a specific position on B-ASO increases the kinetics of enzymatic hydrolysis of complementary RNA more than 30-fold compared with unmodified duplex ASO/RNA by endoribonuclease H (RNase H) [44]. In addition, boron clusters and their conjugates with nucleosides exhibit high binding affinity for plasma proteins both in vitro and in vivo [53]. Barba-Bon et al. showed the interaction of boron clusters with cationic peptides, charged biomolecules (such as acetylcholine and amino acids), vitamins, antibiotics, neuromuscular blockers, and proteins. In addition, the binding affinity of the boron cluster to the lysine amino acid was shown to be stronger than to the arginine amino acid. Moreover, this finding indicates that interactions other than the conventional bonding (i.e., Coulombic attractions, salt bridges, or hydrogen) are important contributors to the cluster–peptide affinity and uptake by the membrane cells [54]. Interestingly, sunitinib decorated with a boron cluster against glioma cancer cells was found to be four times more cytotoxic than unmodified sunitinib. This conjugate was selective and increased the accumulation of boron in F98 glioma cells (overexpressed EGFR) compared to normal mouse astrocytes [55]. Based on the results of our LS-MS/MS analysis (Figure 2E), one could speculate that the increased activity conjugate of the sunitinib–boron cluster (a low molecular weight tyrosine kinase inhibitor) observed in this work was due to the affinity of the boron cluster for the amino acid chain of EGFR (Figure 2E, red bold), thereby increasing the uptake of the agent tested into cells. However, this interesting phenomenon has not been well addressed. Moreover, Couto et al. confirmed that the use of hybrid erlotinib with boron clusters as potential EGFR inhibitors were most effective in U-87 MG and HT-29 cancer cells compared to unmodified erlotinib treatment. Among ionic derivatives, erlotinib with metallacarborane (COSAN) was the most active inhibitor investigated in both EGFR-overexpressing cell types (i.e., U87-MG and HT-29) [56]. In this case, the nature of the boron cluster, in addition to other factors, could have influenced the uptake and high activity of the boron hybrid compounds in EGFR-overexpressing cells.

Next, we investigated the free uptake of B-ASO by different cell types (Figure 3B). Interestingly, the highest level of boron accumulation was observed in A431 cells (27 µg B/g, 5.4 µg ^10^B/g) and U87-MG glioblastoma cancer cells (24 µg B/g, 4.8 µg ^10^B/g), which are characterized by overexpression of the EGFR [51,52,57]. Horiguchi et al. determined that a concentration of 5 ppm of ^10^B (5 µg of ^10^B/g cells) in cells was sufficient for effective BNCT in vivo [48]. Thus, our B-ASO compounds fulfill this criterion and might be promising carriers of boron compounds for BNCT.

In another study, high accumulation of boron compounds was observed in the HT-29 cell line derived from Homo sapiens colorectal adenocarcinomas, which also overexpress EGFR [55]. The boron polymer compounds were successfully maintained at high concentrations in and around subcutaneous C26 tumors in BALB/c mice for 24 h. However, 24 h after intravenous injection of the boron compound, boron concentration in the blood was quite low (1–3 ppm) [58]. Moreover, the boron compounds can be rapidly cleared by the major clearance organs, such as the liver, kidney, and spleen in vivo, to background levels, making BNCT a safer treatment option.

We used ICP MS analysis to monitor delivery of boron compounds (i.e., B-ASOs) to five types of normal cells and macrophages from three donors (Figure 3B). Importantly, our studies have demonstrated minimal uptake of B-ASOs by human fibroblasts (NHDFs, 6 µg B/g cells), human osteoblasts (h-Fob 1.19, 6 µg B/g cells), mouse embryonic fibroblasts (MEF-WT, 8 µg B/g cells), colon epithelial cells (CCD 841, 10 µg B/g cells), kidney cells (HEK-293, 13 µg B/g cells), and macrophages (3 µg B/g cells, donor 3), which are reported as cells with normal and low EGFR expression [36,51]. Viñas et al. confirmed that COSAN was present in excretory organs, such as the kidneys and liver, soon after injection and remained after 1 h. In addition, low accumulation of COSAN was detected in the heart and muscles [59].

Western blot analysis and quantification of EGFR levels confirmed that A431 cells have greater expression of EGFR compared to HeLa, whereas the lowest EGFR levels were observed in the MCF-7 cancer cell line (Figure 3E and Figure S6). The amount of endogenous EGFR levels in the cells correlates with the concentration of boron delivered by B-ASOs, as analyzed by ICP MS (Figure 3A,B,E).

In our previous work, we observed that DNA nanostructures incorporated in the chain boron clusters (1,2-DCDDB) had a low likelihood of penetrating the phospholipid membrane of A431 cells. A concentration of 4 µM of the boron cluster nanostructures delivered 13 µg B/g cells, which corresponds to 2.6 µg ^10^B/g cells [27]. This discrepancy in boron uptake could be due to a different chemical environment of the boron clusters, as reported previously [26]. As a result, the boron clusters in the nanostructures were not available to bind the EGF receptor due to the spatial limitations. In the present work, the tested “fishing rod” (BIOT-1-FESAN or BIOT-1-1,2-DCDDB) and B-ASO have boron clusters attached to the chain of therapeutic nucleic acids.

BNCT is a type of targeted radiotherapy which requires the ratio of boron between tumor and normal tissue (T/N) to be greater than 2.5 [60]. Thus, the T/N ratio is an important parameter used to estimate the boron concentration in the tumor, which is then used to calculate the radiation doses delivered to the tumor and normal tissue. Our in vitro model confirmed that the free uptake of B-ASOs by the A431, U87-MG, and HTC-116 cancer cells lines was ~4.8, 4.3, and 3.2 times higher, respectively, compared to normal cells (NHDF) (C/N). Interestingly, the ratio between the same type of cancer cells and human macrophage cells from three individual donors (C/M) ranged from 11.8 to 17.6 (Figure 3B and Figure S7). These high C/M ratios are very promising. However, more advanced experiments are required which will utilize whole blood rich not only in macrophages but also in other cellular components. Our results regarding the ratio of boron content in cancer cells compared to normal cells (C/N) and cancer cells compared to macrophages (C/M) fulfilled the requirements for studying the uptake of the boron compound, B-ASO, by cells for BNCT, which is currently performed in the Laboratory of Applied Nuclear Energy (L.E.N.A., University of Pavia, Pavia, Italy).

We compared the transfection efficiency of B-ASOs in HeLa cells using commercial Lipofectamine 2000 or without a commercial transfection agent (free uptake) (Figure 3C). B-ASO (80 pmol/well) was taken up by HeLa cells without the transfection agent as efficiently as with Lipofectamine 2000. Moreover, B-ASOs can be efficiently taken up by cancer cells without using a commercial transfection agent, making it a safe and promising boron carrier for future in vivo therapy.

B-ASOs have a dual therapeutic potential against cancer cells: anti-EGFR inhibitory and boron carrier (^10^B) delivery for BNCT which we have described previously [23,24,26,27,44]. Importantly, the ASO sequences of these structures were designed to be effective silencers towards *EGFR* mRNA, as well as EGFR variant III mRNA [14,61]. We observed that B-ASO (80 pmol) can be freely taken up into A431 cells and mediated by FESAN-EGFR. Furthermore, after 48 h of incubation, ~70% of exogenous EGFR-GFP protein was silenced compared with ASO-C (non-active ASO) (Figure 3F). Previously, the greatest silencing of *EGFR* gene activity by B-ASO in HeLa cells was observed at concentrations above 100 nM (via Lipofectamine 2000) and resulted in a 40% decrease in EGFR-GFP protein levels determined using DFA [24]. In this study, we have shown that B-ASO is efficiently taken up by EGFR-FESAN in EGFR-overexpressing cancer cells and has higher anti-EGFR silencing activity than it has in case of delivery by the commercial Lipofectamine 2000 transfection agent.

Boron clusters (C_2_B_10_H_12_) are used as hydrophobic core structures in various bioactive compounds, especially as ligands of various nuclear receptors, such as retinoid receptors [62], androgen receptors [63], vitamin D receptors [64], ghrelin receptors [65], neuropeptide Y receptors [66], adenosine receptors [67], HIV protease receptor ligands, 5-lipoxygenase receptor ligands, cyclooxygenase ligands, delocalized lipophilic cation ligands, hyaluronic acid ligands, and histone deacetylase ligands [47]. Interestingly, progesterone receptors are also known as being associated with cancer, but they have proven difficult to target. Mori et al. developed a new class of boron cluster agents that target this receptor which causes many problems with selectivity binding in steroid-based therapies. The authors confirmed that the para-isomer of carborane exhibited 10-fold greater antagonistic activity than the other carborane derivatives, indicating high binding affinity to the hydrophobic pocket of PR and low IC_50_ values [68].

The cellular uptake patterns of different carboranes or boron cages are varied. Chou et al. reported that the accumulation of sodium borocaptate (BSH) in HepG2 cells is time-dependent and reached 23 µg/g 4 h post-incubation [69]. BSH uptake by the tumor cells is believed to mainly rely on passive diffusion [70]. In contrast to how small molecules are transported into cells, nanoparticles (NPs) can be internalized to the cytoplasm via macropinocytosis or phagocytosis [71,72,73,74].

Recently, Barba-Bon and colleagues performed experiments using vesicles which mimic biological membranes to transport anionic boron clusters. They found that the di-anionic boron cluster ([B_12_Br_12_]^2−^) not only retained its transport activity in the vesicle membranes, but also largely outperformed the mono-anionic compound which was used as a reference. Despite the excellent transport profile of the boron cluster ([B_12_Br_12_]^2−^), the anionic membranes were not perturbed even at concentrations an order of magnitude higher than those required for transport activity. Therefore, the observed transport phenomena and the cargo scope in vesicle transport experiments are unique [54]. Interestingly, the boron cluster derivatives, COSAN, exhibited significant and strong translocation through lipid bilayer membranes using supramolecular tandem membrane assays that enable real-time monitoring of their translocation with fluorescence [75]. Grüner et al. and Viñas et al. also developed and investigated a series of metallacarborane compounds (COSAN) and found them to be effective in membrane crossing tests both in vitro and in vivo [76,77]. The observed increase in membrane penetration efficiency, combined with evidence of specific interactions with amino acids, underlines the importance of metallacarborane as a 3D entity capable of a variety of interactions ranging from hydrophobic to dihydrogen bonding [76,77]. Interestingly, cellular uptake of metallacarboranes in HeLa cells was 1000-fold more efficient than that of *closo*-dodecaborates [78].

In addition, EGFR functions were blocked in A431 and U87-MG cells to confirm the binding and uptake of B-ASO by EGFR into the cells. Blocking the extracellular domain of EGFR using an EGF ligand or downregulation of the *EGFR* mRNA with ASO anti-EGFR reduced the B-ASO uptake by more than 50% in the tested cancer cell lines. We confirmed that the EGF receptor is involved in the uptake of boron cluster compounds from the extracellular medium. Surprisingly, despite blocking EGFR activity, the B-ASO compounds still entered cells via a different pathway, which was confirmed using ICP MS analysis [54]. In addition, HeLa, MG-63, and MCF-7 cancer cells, which have low EGFR overexpression compared to normal cells, took up the B-ASO compound at similar, low levels (Figure 3B).

In the present study, we assessed the kinetic capacity of the free uptake of FAM-B-ASO or FAM-1-FESAN at a concentration of 80 pmol/well by A431 cells. After 1–3 h of incubation with the FAM-1-FESAN compound, a dense field of punctate green fluorescent vesicles along the cell membrane, suggesting endocytosis via the membrane receptor (possibly EGFR), was observed (Figure 5A,B and Figure S8). The time course of FAM-B-ASO or FAM-1-FESAN uptake is consistent with the EGFR recycling time of ca. 0.5 h [79,80]. Other studies confirmed that incubation times longer than 1 h resulted in the granular pattern of the tested compound, which was more pronounced in A431 endo/lysosomes [81]. After 0.5 h of incubation with FAM-B-ASO and FAM-1-FESAN, we observed strong uptake, 40% and 50% by A431, respectively, compared to control (compounds without cells). After 12 h of incubation with FAM-B-ASO and FAM-1-FESAN, the fluorescence level of the tested boron compounds was ~70% in A431 cells (Figure 5D). The time course results are consistent with ICP MS measurements (Figure 5E) and fluorescence microscopic analysis (Figure 5C). In BNCT, a simple and single application of boron compounds during treatment and their rapid uptake by cancer cells is key. We confirmed the feasibility of these performance requirements in this experiment.

The location of the boron clusters in tumor cells plays an important role in the success of targeted BNCT. FAM-B-ASO or FAM-1-FESAN was predominantly localized in the membranes of the endoplasmic reticulum (Figure 5C). In previous work, we performed MST analysis that demonstrated the strong affinity of free FESAN to ssDNA [25]. The affinity of boron clusters for nucleic acids is a key factor for BNCT which causes direct DNA damage via single-stranded breaks (SSBs) and double-stranded breaks (DSBs) through ionization [18]. A similar observation was confirmed by Viñas and coworkers, who demonstrated that metallacarboranes (COSAN) are localized primarily in the membranes but also present in the cytoplasm, nucleus, and cytoskeleton of U87-MG and A431 cells [59]. Another recent study in A431 cells showed that EGFR binds phosphorothioate-modified ASOs (PS-ASOs) at the cell surface. This binding is important for efficient PS-ASO uptake via trafficking from early to late endosomes and may contribute to the release of PS-ASOs from late endosomes [79]. Therefore, unmodified B-ASOs were used in the current work to avoid chemical modification disruptions which could affect the efficiency of B-ASO penetration into cells overexpressing EGFR. Indeed, using pull-down analyses we demonstrated that the BIOT-1 oligomer (2′-OMe), without any boron cluster decoration, has low affinity for cellular proteins and does not bind to any proteins (including EGFR). Because other studies have shown that 2′-OMe modifications are able to attenuate adverse effects and thus increase the tolerability and therapeutic index of drugs [82], we did not use 2′-OMe ASO in our biological analysis to avoid interfering with B-ASO uptake. However, in future research, B-ASO will contain chemical modifications and ligands to ensure nucleolytic stability of oligonucleotides.

The physicochemical nature of the boron clusters prohibited investigation of the endosomal pathway of FAM-B-ASO. Boron clusters have a high affinity for various proteins (cellular, albumin, enzymes, or receptors), lipids, or cyclodextrins. After lysosome staining, we observed overlapping fluorescence spectra and non-specific staining. In addition, FESAN with iron (Fe^3+^) cations exhibited Cyanine3 (Cy3) fluorescence quenching by the Förster Resonance Energy Transfer (FRET) (Figure S10) [83]. Other studies confirmed the ability of boron clusters to generate overlapping fluorescence spectra and difficulties in analysis [53,54]. Using commercial dyes and antibodies to assess the endosomal pathway would interfere with FAM-B-ASO uptake mediated via EGFR-FESAN. Therefore, a FAM-1-FESAN probe was used to assess the free uptake of B-ASO by EGFR-overexpressing cancer cells.

It is worth mentioning that B-ASOs do not reduce mitochondrial activity in cancer cells under in vitro conditions, as we have shown in our previous work [23,24]. In addition, Goszczyński and co-authors have shown that compounds containing boron clusters (thymosin β4-anionic boron cluster) are not cytotoxic to cardiomyocytes and even improve the viability of these cells [84]. It has also been shown that RNA aptamers decorated with boron clusters have high efficacy in BNCT glioma cancer cells, while normal cells are not sensitive and survive irradiation [85]. Compounds containing boron clusters are commonly used in BNCT in vivo models [86]. The problem faced by researchers is to achieve high selectivity and uptake of boron compounds by the tumor mass. Interestingly, boron compounds did not show systemic toxicity in mice and increased the apoptosis rate upon irradiation with thermal neutrons [87]. In addition, boron clusters have been shown to cross the blood-brain barrier (BBB) under in vitro [88] and in vivo studies [89].

The results presented here demonstrate that the level of EGFR expression correlates with the free uptake of B-ASO by tumor cells and provides for high selectivity compared to normal cells. Therefore, these results suggest a new delivery system for ASO and other drugs coupled to boron clusters. To ensure successful delivery and treatment of the novel ASO delivery system, prior quantification of EGFR expression is needed to enable a more specific selection of tumors cells in the patient’s body that will benefit from B-ASO in BNCT and EGFR inhibition dual therapy.

## 4. Materials and Methods

Chemicals were obtained from Aldrich Chemical Company (St. Louis, MO, USA) and were used without further purification unless otherwise stated. Unmodified nucleoside phosphoramidites, 1 dimethoxytrityloxy-2-(N-biotinyl-4-aminobutyl)-propyl-3-O-(2-cyanoethyl)-(N,N-diisopropyl)-phosphoramidite, and 5′ -dimethoxytrityl-2′-propargyluridine 3′-O-(N,N-diisopropyl-2-cyanoethyl) phosphoramidite were purchased from ChemGenes Corporation (Wilmington, MA, USA). Additionally, 2 dimethoxytrityloxymethyl-6-(3′,6′-dipivaloylfluorescein-6-yl-carboxamido)-hexyl-1-O-[(2-cyanoethyl)-(N,N-diisopropyl)]-phosphoramidite was purchased from Glen Research (Davis Drive, Sterling, VA, USA). Also, 1,2-dicarba-closo-dodecaborane and FESAN were obtained from Katchem (Rež n/Prague). C18 SepPak cartridges were purchased from Waters Corp., (Miliford, MA, USA), and ammonium hydroxide (30%) (J.T. Baker brand) was obtained from Avantor Performance Materials (Center Valley, PA, USA).

Negative ion MALDI mass spectra were recorded on an Axima Performance (Shimadzu, Kyoto, Japan) instrument equipped with a nitrogen laser (337 nm) in the linear mode. A mixture of a 50 mg/mL solution of 3-hydroxypicolinic acid in 50% acetonitrile and a 50 mg/mL solution of diammonium hydrogen citrate in deionized water (8:1, *v*/*v*) was used as a matrix.

Negative ion electrospray mass spectra (ESI-MS) were recorded on a Synapt G2 Si high-resolution mass spectrometer (Waters) equipped with a quadrupole time-of-flight mass analyzer (Waters Corp., Miliford, MA, USA). Raw data were collected in the continuum resolution mode and deconvoluted using the MaxEnt1 algorithm to a zero-charge state mass.

All UV absorption measurements were performed in 1 cm path length cells using a Jasco V 770 UV−VIS/NIR spectrophotometer. Solutions of the compounds for UV experiments were prepared by dissolving each compound in deionized water, and the measurements were performed at ambient temperature.

### 4.1. Synthesis of Oligonucleotides

Synthesis of substrate oligonucleotides (all non-modified compounds and modified with 2′-propargyl-uridine moiety, Table S1) was performed according to a standard procedure, by the use of the phosphoramidite solid-phase method, an LCA CPG glass support, and commercially available nucleoside phosphoramidites. Synthesis at a 0.1 µmol scale was performed on an H6 GeneWorld automated DNA/RNA synthesizer (K&A, Laborgeraete GbR, Schaafheim, Germany) under the conditions recommended by the manufacturer.

After synthesis, all compounds were cleaved from the solid support under standard conditions, purified by reverse-phase HPLC and desalted on C18 SepPak cartridges. Also, subsequent removal of the 5′-DMTr groups from oligonucleotides, which were not labelled with 6-FAM residue, was performed on C18 SepPak cartridges by treating the adsorbed oligomers with 2% TFA, followed by washing the cartridges with water (approximately 10 mL) and eluting the desired product with 30–50% CH_3_CN in water (approximately 2.0 mL). The proper sequence and the purity of the compounds were confirmed by ESI-Q-TOF/MALDI-TOF MS.

### 4.2. Synthesis and Purification of Oligonucleotides Modified with Boron Cluster

Oligonucleotides possessing 2′-*O*-propargyl uridine moiety (Table S1) were post-synthetically modified with boron clusters using a copper-catalyzed azide-alkyne cycloaddition reaction, performed according to the standard copper sulfate procedure [Glen Research, Sterling, VA, USA. Available online: (Glen Report 25.24 - New Products - TEMPO Spin Labels for Click Chemistry and 5-Ethynyl-dU (glenresearch.com), accessed on 18 May 2017 [90])] and in our previous papers [23,24].

Azides derivatives of 1,2-dicarba-*closo*-dodecaborane (N_3_-alkyl-1,2-DCDDB, [C_2_B_10_H_12_]) (Figure S1) and [(3,3′-Iron-1,2,1′,2′-dicarbollide)^−^] (N_3_-alkyl-FESAN), [Fe(C_2_B_9_H_11_)_2_]^−^ (Figure S2) used in these reactions were synthesized according to the procedures available in the literature [42,43,44].

The oligonucleotides modified with boron clusters were isolated from reaction mixtures by reverse-phase high-performance liquid chromatography (RP-HPLC) with buffer A (0.1 M CH_3_COONH_4_/H_2_O) and buffer B (100% CH_3_CN) at a 1 mL/min flow rate. The buffer B gradient in buffer A was as follows: (1) 0–2 min 0% buffer B; (2) 2–25 min 0–70% buffer B; (3) 25–30 min 70% buffer B; (4) 30–35 min 70–0% buffer B; and (5) 35–38 min 0% buffer B. The collected oligonucleotide fractions were desalted on C18 SepPak cartridges. The molecular mass of all the synthesized oligonucleotides was confirmed by ESI-Q-TOF/MALDI-TOF mass spectrometry and their purity by RP-HPLC.

### 4.3. RP-HPLC Analysis

RP-HPLC analyses were performed on a Shimadzu Prominence HPLC system (Kyoto, Japan) using a Kinetex 5 µm C-18 column (100 Å, 250 mm × 4.6 mm column, Phenomenex). All analyses were performed at ambient temperature. UV detection was performed at λmax 260 nm and λmax 494 nm, and the amount of compound was defined in optical units (OD) at the 260 nm wavelength.

### 4.4. Pull-Down Assay Using Biotinylated BIOT-1 Probe Conjugated with Boron Clusters (FESAN or 1,2-DCDDB)

First, 28.5 nmols (1.5 OD) of compounds (BIOT-1-FESAN or BIOT-1-1,2-DCDDB) were added to 0.6 mL high capacity streptavidin agarose resin (Thermo Scientific) without 50% DMSO (removed DMSO by washing 6 times with PBS) and incubated for 2 h (room temperature, gently mixing, total volume 1 mL). The A431 or MEF-WT cells protein lysate was prepared in the NP-40 buffer containing 50 mM Tris pH 8.0, 150 mM NaCl, 0.5% IGEPAL and supplemented with 1% protease inhibitors cocktail (Roche). Next, the beads with immobilized compounds BIOT-1-FESAN or BIOT-1-1,2-DCDDB were added to the A431 or MEF-WT cell protein lysate (5 mg of total protein) and incubated for 2 h at 4 °C with gentle mixing. As a control, A431 or MEF-WT cells protein lysate incubated with the streptavidin agarose beads BIOT-1 without boron cluster was used. After incubation, the beads were centrifuged (2000 rpm, 2 min, 4 °C) and washed 6 times with 1 mL of NP-40 buffer (50 mM Tris pH 8.0, 150 mM, NaCl, 0.5% IGEPAL). After the last wash, the beads were resuspended in the buffer with SDS buffer and denatured for 10 min at 95 °C. Released proteins (30 µL/well) were separated by SDS-PAGE (4% stacking, 7% resolving gel) and silver stained. Bands of interest were cut out of the gel and digested with trypsin. Tryptic peptides were separated by liquid chromatography and analyzed by LC-MS/MS with an Orbitrap mass spectrometer (Thermo Fisher Scientific). The sequences of the LC-MS/MS spectra were identified by correlation with the human protein sequence database (Swiss-Prot protein database) using MASCOT software version 1.9 (http://www.matrixscience.com/ (accessed on 18 December 2020). The significance threshold was *p* < 0.05 and ion score or expected cut-off was 46. Experiments were performed independently in triplicate.

### 4.5. Cell Lines and Culture Conditions

HeLa (human cervical carcinoma) cell lines (ATCC, Manassas, VA, USA) cells were cultured in RPMI 1640 medium (Gibco, BRL, Paisley, New York, NY, USA) supplemented with 10% heat-inactivated fetal bovine serum (FBS) (Gibco, BRL, Paisley, New York, NY, USA), 100 U/mL penicillin, and 100 µg/mL streptomycin (Gibco, BRL, Paisley, New York, NY, USA) at 37 °C and 5% CO_2_. The A431 (human epidermoid squamous carcinoma), MCF-7 (human breast cancer), and U87-MG (human glioblastoma astrocytoma) cell lines (ATCC, Manassas, VA, USA) and primary normal human dermal fibroblasts (NHDF), isolated from the dermis of juvenile foreskin cell line (Celther, Poland), were cultured in DMEM containing 4.5 g/L D-glucose, 0.11 g/L sodium pyruvate and without L-glutamine (Gibco, BRL, Paisley, New York, NY, USA) supplemented with 10% (15% for foreskin fibroblast cell line) heat-inactivated fetal bovine serum (FBS) (Gibco, BRL, Paisley, New York, NY, USA), 100 U/mL penicillin, and 100 µg/mL streptomycin (Gibco, BRL, Paisley, New York, NY, USA) at 37 °C and 5% CO_2_. The HTC-116 (human colorectal carcinoma) cell line (ATCC, Manassas, Virginia, USA) was cultured in McCoy’s 5a Modified Medium containing 1.5 mM L-glutamine and 2200 mg/L sodium bicarbonate (ATCC, Manassas, Virginia, USA) supplemented with 10% heat-inactivated fetal bovine serum (FBS) (Gibco, BRL, Paisley, New York, NY, USA), 100 U/mL penicillin, and 100 µg/mL streptomycin (Gibco, BRL, Paisley, New York, NY, USA) at 37 °C and 5% CO_2_. The MG-63 (human osteosarcoma), and HEK-293 (human embryonic kidney), MEF-WT (mouse embryonic fibroblasts), and CCD-841 CoN (human normal colon) cell lines (ATCC, Manassas, VA, USA) were cultured in (EMEM) containing non-essential amino acids, 2 mM L-glutamine, 1 mM sodium pyruvate, and 1500 mg/L sodium bicarbonate (ATCC, Manassas, VA, USA) supplemented with 10% heat-inactivated fetal bovine serum (FBS) (Gibco, BRL, Paisley, New York, NY, USA), 100 U/mL penicillin, and 100 µg/mL streptomycin (Gibco, BRL, Paisley, New York, NY, USA) at 37 °C and 5% CO_2_. The h-FOB 1.19 (human osteoblast) cell line (ATCC, Manassas, Virginia, USA) was cultured in Ham’s F12 Medium Dulbecco’s and Modified Eagle’s Medium, with 2.5 mM L-glutamine (without phenol red) containing geneticin 0.3 mg/mL G418 (ATCC, Manassas, VA, USA) supplemented with 10% heat-inactivated fetal bovine serum (FBS) (Gibco, BRL, Paisley, New York, NY, USA), at 34 °C and 5% CO_2_. Cell cultures were analyzed for mycoplasma contamination with the EZ-PCR Mycoplasma Detection Kit (BI, Cromwell, CT, USA) and proved negative.

### 4.6. Determination of the Boron Content in Cancer and Normal Cells by ICP MS Measurements

Culture cells were prepared as described above. Twenty-four hours before the experiment, cells were plated in 96-well plates (Perkin-Elmer, Waltham, MA, USA) at a density of 15 × 10^3^ cells per well in a volume of 100 µL of the full medium. After that, for assessment of the free uptake of B-ASO, the medium was replaced with basic medium without FBS in a volume of 100 µL (respectively for each line which was described above) and the aqueous solutions of B-ASO (20, 40, 80 pmol/well) were added to the basic medium and the cells were incubated for 12 h at 37 °C (34 °C for h-FOB 1.19 cell line) in an atmosphere of 5% CO_2_. After 12 h of incubation at 37 °C in an atmosphere of 5% CO_2_, upon medium removal, the cells were treated with trypsin (1.5 h, 20 µL/well, Gibco, trypsin-EDTA (0.5%, no phenol red) and diluted with ultrapure water (18 MΩ·cm, Simplicity Water Purification Systems, Millipore SAS, Molsheim, France) up to a 1.5 mL volume, and then, the ICP MS measurements were performed and data were collected. The boron content was determined with a Varian 810-MS inductively coupled plasma mass spectrometer (Varian, Palo Alto, CA, USA) equipped with a Micromist nebulizer, quartz Scott spray chamber (3 °C), platinum sampler cone, and nickel skimmer cone. The operating parameters were as follows: RF power: 1.4 kW; plasma flow (argon): 17 L/min; auxiliary flow (argon): 1.7 L/min; nebulizer flow (argon): 1.00 L/min; pump rate: 4 rpm; sheath gas (argon): 0.2 L/min; and number of scans: 10, m/z: 10 and 11.

Cell transfection was performed using Lipofectamine 2000 transfection reagent (Invitrogen, New York, NY, USA) at a 2:1 ratio (2 µL of Lipofectamine 2000 per 1 µg of oligonucleotide) according to the manufacturer’s protocol. The calibration curve method was applied. A series of 10 calibration solutions were prepared in the boron concentration range of 0.1–50 µg/L by appropriate dilution of the standard solution of boron (1000 mg/L, Merck, Darmstadt, Germany) using ultrapure water. A linear model of the calibration curve was adopted, with a minimum correlation coefficient of 0.999. The obtained results were the average of concentrations calculated from the calibration curves obtained for both stable boron isotopes (^10^B and ^11^B). Experiments were performed in triplicate and the mean with standard deviation (±SD) was reported.

### 4.7. Human Primary Monocyte-Derived Macrophages and Measurement of Free Uptake B-ASO by Using ICP MS Analysis

Peripheral blood mononuclear cells were isolated from leukocyte-rich buffy coats provided commercially by the Blood Transfusion and Donation Centre obtained from the Regional Blood Transfusion Centre in Lodz, Poland. The blood donors agreed to donating the buffy coat randomly for research purposes during blood donation, maintaining their anonymity.

Monocytes adhering to the plastic were selected and differentiated into macrophages in a macrophage-SFM medium (Gibco, Thermo Fisher Scientific) supplemented with 10 ng/mL granulocyte-macrophage colony-stimulating factor (GM-CSF, Immunotools GmbH, Friesoythe, Germany) and antibiotics, as described previously [91,92]. On day six, the cells were washed twice with PBS, supplied with fresh RPMI 1640 medium (without FBS) supplemented with L-glutamine and antibiotics, and primed with 1 μg/mL lipopolysaccharide (LPS) (InvivoGen, San Diego, CA, USA) for 4 h to induce the expression of the nucleotide-binding oligomerization domain and leucine-rich repeat-containing receptor family pyrin domain-containing 3 (NLRP3) inflammasome components and Interleukin-1β (IL-1β).

Cells were plated in 48-well plates (Perkin-Elmer, Waltham, MA, USA) with 500 µL of the full medium and allowed to incubate for 24 h. Then, the medium was replaced with 200 µL of basic medium without FBS. Aqueous solutions of B-ASO (20, 40, 80 pmol/well) were added to the basic medium, and the cells were incubated for 12 h at 37 °C and 5% CO_2_ to assess free uptake of B-ASO.

After 12 h of incubation at 37 °C and 5% CO_2_, the medium was removed. The cells were then incubated at 4 °C and counted (15 × 10^3^ cells per well). Then, cells were treated with 20 µL trypsin/well for 1.5 h. Afterward, cells were diluted with ultrapure water (up to 1.5 mL for ICP MS analysis which was performed as above). The experiment was carried out using primary cells derived from three independent donors for each concentration and the mean with standard deviation (±SD) was reported.

### 4.8. Dual Fluorescence Assay (DFA) of Silencing Activity of B-ASOs

Construction of the plasmid encoding the EGFP-EGFR fusion gene and its use in DFA experiments performed using cancer cells was described and tested in our previous work [23,24,26,28]. Cells were cultured as described above. Two plasmids, pEGFP-EGFR and pDsRED-N1, were transfected using Lipofectamine 2000. After 6 h of incubation, the transfection mixture was replaced with 200 µL of fresh DMEM with antibiotics (without FBS) per well. Next, 80 pmol of the tested oligonucleotides (B-ASO, ASO anti-EGFR, and ASO-C) were added to each well without commercial transfectant for 48 h at 37 °C and 5% CO_2_ atmosphere. Then, the cells were washed twice with PBS buffer (without Ca^2+^ and Mg^2+^) and lysed with NP-40 buffer (150 mM NaCl, 1% IGEPAL, 50 mM Tris-HCl pH 7.0 and 1 mM PMSF) overnight at 37 °C. The fluorescence of the prepared cell lysates was measured using a Synergy HT reader (BIO-TEK, Waltham, MA, USA). Three independent experiments were performed.

### 4.9. Microscopic Analysis of the FAM-B-ASO and FAM-1-FESAN Uptake by Cancer and Normal Cells

A431 cells and NHDFs were cultured as described above. Cells were plated in 96-well plates at a density of 15 × 10^3^ cells per well with 100 µL of the full medium and allowed to incubate for 24 h. Then, the medium was replaced with 100 µL of basic medium without FBS. Aqueous solutions of FAM-B-ASO or FAM-1-FESAN (20, 40, 80 pmol/well) were added to the basic medium and the cells were incubated for 12 h at 37 °C and 5% CO_2_.

After 11.5 h of incubation, the medium was mixed with 5 µg/mL DAPI dye (Invitrogen, Thermo Fisher Scientific, MA, USA) and 1 µmol/mL ER-Tracker Red dye (Invitrogen, Thermo Fisher Scientific, MA, USA) to stain cell nuclei blue and ER membranes red, respectively. The cells were incubated with the dyes for 30 min in the dark at 37 °C. After incubation, the cells were washed twice with PBS buffer (with Ca^2+^ and Mg^2+^) and imaged under a fluorescence microscope (Nikon-Eclipse, Tokyo, Japan) with DAPI at λ_ex_ = 340–380 nm (λ_DM_ = 400 nm, λ_BA_ = 435–485 nm) and FITC at λ_ex_ = 465–495 nm (λ_DM_ = 505 nm, λ_BA_ = 515–555 nm), B-2A (longpass, λ_ex_ = 450–490 nm(λ_DM_ = 505 nm, λ_BA_ = 520 nm), TX red (λ_ex_ = 540–580 nm, λ_DM_ =595 nm, λ_BA_ = 600–660 nm), and G-2A longpass λ_ex_ = 510–560 nm(λ_DM_ = 575 nm, λ_BA_ = 590 nm), where DM is a dichroic mirror and BA is an absorption filter. Three independent experiments were performed.

### 4.10. Reduction and Blocking of EGFR in Cancer Cells

Cells were cultured as described above. Cells were seeded in a 96-well plate at a density of 15 × 10^3^ cells per well with 100 µL of full medium and incubated for 24 h.

(I) After 24 h of incubation, the cells should be 80% confluent. Directly before the transfection, the cell media was replaced with antibiotic-free medium (100 µL per well). Cells were transfected using the Lipofectamine 2000 transfection reagent at a 2:1 ratio (2 μL of Lipofectamine 2000 per 1 μg of nucleic acid ASO anti-EGFR or ASO-C at 200nM) according to the manufacturer’s protocol. After 5 h of incubation, the transfection mixture was replaced with 200 µL of fresh full medium. After 36 h of incubation, the media was replaced with 100 µL basic media (without FBS) containing aqueous solutions of B-ASO (80 pmol/well). Then, the cells were incubated for 12 h at 37 °C and 5% CO_2_. After 12 h of incubation, the media was removed. The cells were treated with 20 µL of trypsin per well for 1.5 h. Then, the cell was diluted with ultrapure water up to 1.5 mL volume for ICP MS analysis which was described above.
(II) After 24 h, the full medium was mixed with EGF (final concentration 10 ng/mL) and incubated for 1 h at 37 °C and 5% CO_2_. Then, the mixture was replaced with 100 µL basic media (without FBS) containing B-ASO (80 pmol/well), and the cells were incubated for 12 h at 37 °C and 5% CO_2_.

After 12 h of incubation, the media was removed. Then, the cells were treated with 20 µL of trypsin for 1.5 h and diluted with ultrapure water up to 1.5 mL for ICP MS analysis. Experiments were performed in triplicate and the mean with standard deviation (±SD) was reported.

### 4.11. Time Course Analysis of Cellular Uptake of FAM-B-ASO and FAM-1-FESAN

Cells were cultured as described above. Cells were seeded in a 96-well plate at a density of 15 × 10^3^ cells per well with 100 µL of full medium and incubated for 24 h.

Then, the media was replaced with 100 µL basic media (without FBS) containing aqueous solutions of FAM-B-ASO or FAM-1-FESAN (80 pmol/well). The cells were incubated for 0.5, 1, 3, 6, and 12 h at 37 °C and 5% CO_2_. FAM-B-ASO and FAM-1-FESAN (80 pmol/well) and itself were used as a control (compounds without cells). After incubation, the cells were washed twice with PBS buffer (containing Ca^2+^ and Mg^2+^) and lysed with NP-40 buffer (150 mm NaCl, 1% IGEPAL, 50 mm Tris-HCl Ph 7.0, 1 mm PMSF) overnight at 37 °C. The prepared cell lysates were used for fluorescence measurements. The green fluorescence values of FAM-B-ASO or FAM-1-FESAN were determined using a Synergy HT reader (BIO-TEK). Data were quantified with KC4 software. The excitation and emission wavelengths for each protein were as follows: λ_Ex_ = 485/20 and λ_Em_ = 528/20 nm. Experiments were performed in triplicate and the mean with standard deviation (±SD) was reported.

### 4.12. Western Blot Analysis of EGFR Protein Level in A431, HeLa and MCF-7 Cells

A431, HeLa, and MCF-7 cells were cultured as described above. Cells were cultured for 48 h on 25 cm^3^ flasks (PerkinElmer) to reach 80% confluence. After the incubation period ended, cells were washed with cold PBS and lysed with 99 µL of RIPA buffer and 1 µL of 1% protease inhibitor (100×). Samples were incubated for 30 min at 4 °C and centrifuged for 15 min at 14,000 rpm at 4 °C. Protein concentration was determined using the Bradford protein assay (BioRad, Hercules, CA, USA). Thirty micrograms of protein per well were separated on SDS PAGE (4% and 8%; pH is 6.8 or 8.8, respectively) reduction gradient gels and transferred to nitrocellulose membranes (Thermo Scientific, Waltham, MA, USA) for semi-dry Western blot analysis. After blocking nonspecific binding sites, the membranes were incubated overnight at 4 °C with primary antibodies: anti-ACTIN Mab (1:500) mouse monoclonal antibody, clone C4 (Abnova, Taiwan), and anti-EGFR A10 (1:200) mouse monoclonal antibody (Santa Cruz Biotechnology, Heidelberg, Germany).

Membranes were washed three times for 20 min at room temperature before incubation with a secondary antibody for 1 h at 4 °C. The secondary antibody was goat anti-mouse IgG (1:5000) (H&L) peroxidase (Abnova, Taiwan). Membranes were scanned using an ELC Western blot Pierce blotting substrate (Sigma Aldrich, Oakville, ON, Canada) for 5 min in darkness and analyzed using a G-Box apparatus (Syngene, Cambridge, UK). EGFR protein levels relative to actin levels (100%) in A431, HeLa, and MCF-7 cancer cells and immunoblot quantification were measured using G-BOX. Experiments were performed in duplicate and the mean with standard deviation (±SD) was reported.

### 4.13. Statistical Analysis

The statistical analyses were carried out for the results shown in Figure 3B,F and Figure 4A. At least three independent experiments were performed, unless otherwise stated. The differences between three independent groups of data were calculated using the parametric Student’s *t*-test. To compare the means of three or more groups, one-way ANOVA and post-hoc Tukey honest significant difference (HSD) test (indicating an overall statistically significant difference in the group means) were used. The HSD test was conducted to confirm where the differences occurred between groups. The homogeneity of variances was verified by Levene’s test and the Brown–Forsythe test. All statistical analyses were carried out using Statistica ver. 8.0 software (StatSoft Inc., Tulsa, OK, USA). A value of *p* < 0.05 was considered statistically significant.

## 5. Conclusions

In this work, we focused on antisense oligonucleotides decorated with boron clusters (FESAN), known as B-ASOs, which have dual therapeutic potential against cancer cells—they may act as inhibitors of *EGFR* gene expression and boron carriers for BNCT. We designed molecules based on a short fragment of a DNA oligonucleotide conjugated with boron clusters and a biotin moiety as a “fishing rod with boron cluster bait” to identify proteins to which these molecules have high binding affinity. These fishing rods bound (among others) to EGFR protein present in protein lysates from EGFR-overexpressing A431 cancer cells. Moreover, the tested B-ASO anti-EGFR was efficiently introduced into A431 cells (without using a routine transfection agent), resulting in sufficient boron accumulation and downregulation of EGFR. The high free uptake of boron compounds by A431 cells compared with normal cells was demonstrated by fluorescence imaging and inductively coupled plasma mass spectrometry (ICP MS). The satisfactory ratio of boron content in cancer cells (A431) to normal cells (NHDF) (4.8:1) was assessed. The data obtained suggest that nucleic acid fragments decorated with boron clusters can be freely taken up by EGFR-overexpressing cancer cells via EGF receptor-mediated endocytosis.

This discovery is of great importance as a new delivery system for ASOs and other drugs coupled to boron clusters and opens a new area for antisense and radiation-based cancer therapy, which may be termed B-ASO-BNCT-Oncology (B.A.B.O.).

## Figures and Tables

**Table 1:** Sequences, spectral (MS), and chromatographic (RP-HPLC) data of oligonucleotides modified (1) with: biotin (BIOT), uridine-boron clusters (**U_B.1_**-FESAN, **U_B__.__2_**-1,2-DCDDB), or 6-carboxyfluorescein (FAM).

No.	Sequences	MW calc. (g/mol)	MALDI-TOF MS (*m/z*)	RP-HPLC (Rt, min)
**BIOT-1-FESAN**	5′-biotin-2′OMe(GAU_B.1_UC)-3′	2507.4	2509.8	20.3 *
**BIOT-1-1,2-DCDDB**	5′-biotin-2′OMe(GAU_B.2_UC)-3′	2283.6	2286.7	17.7 *
**BIOT-1**	5′-biotin-2′OMe(GAUUC)-3′	2034.5	1016.2 ^1^/ 2032.4 ^2^	15.5
**FAM-1-FESAN**	5′-FAM-2′OMe(GAU_B.1_UC)-3′	2610.9	2611.8	20.2 *
**ASO anti-EGFR**	5′-d(TTT CTT TTC CTC CAG AGC CCGA)-3′	6612	6612	16.0
**ASO-C**	5′-d(ATG AAG GTT CAA TCT GAT TTT)-3′	6450.3	6450.12	12.8
**B-ASO**	5′-d(U_B.1_TU_B.1_ CU_B.1_TTU_B.1_CCU_B.1_C CAG AGC CCGA)-3′	9063.0	9065.8	24.7 *
**FAM-B-ASO**	5′-FAM-d(U_B.1_TU_B.1_CU_B.1_TTU_B.1_CCU_B.1_C CAG AGC CCGA)-3′	9601.7	9603.0	24.0 *

^1^ double charge ions; ^2^ after deconvolution; * HPLC program using 100% ACN as buffer B.

## Data Availability

Not applicable.

## Supplementary Materials

The following supporting information can be downloaded at: https://www.mdpi.com/article/10.3390/ijms232314793/s1.
