# Correction: Kaniowski et al. EGFR-Targeted Cellular Delivery of Therapeutic Nucleic Acids Mediated by Boron Clusters. *Int. J. Mol. Sci.* 2022, *23*, 14793

**DOI:** 10.3390/ijms27156551

**Published:** 2026-07-23

**Authors:** Damian Kaniowski, Justyna Suwara, Katarzyna Ebenryter-Olbińska, Agata Jakóbik-Kolon, Barbara Nawrot

**Affiliations:** 1 Centre of Molecular and Macromolecular Studies, Polish Academy of Sciences, Sienkiewicza 112, 90-363 Lodz, Poland; 2 Department of Inorganic, Analytical Chemistry and Electrochemistry, Faculty of Chemistry, Silesian University of Technology, Krzywoustego 6, 44-100 Gliwice, Poland

In the original publication [1], there was a mistake in the Supplementary Materials. A Supplementary Information file unrelated to the manuscript was attached due to an inadvertent error. The correct Supplementary Information file has now been provided.

The correct Supplementary Materials can be downloaded at https://www.mdpi.com/article/10.3390/ijms232314793/s1.

The authors confirm that the scientific conclusions remain unaffected. This correction was approved by the Academic Editor. The original publication has also been updated.

## References

[1] Kaniowski D., Suwara J., Ebenryter-Olbińska K., Jakóbik-Kolon A., Nawrot B. (2022). EGFR-Targeted Cellular Delivery of Therapeutic Nucleic Acids Mediated by Boron Clusters. Int. J. Mol. Sci..

