# Supplementary material for: EGFR-Targeted Cellular Delivery of Therapeutic Nucleic Acids Mediated by Boron Clusters
Source: Int J Mol Sci. 2022 Nov 26;23(23):14793. doi: 10.3390/ijms232314793 (PMC9740766; doi:10.3390/ijms232314793)
Supplement: Supplementary file 1 [file ijms-23-14793-s001.zip › ijms-2005970-supplemental-highlight.pdf]

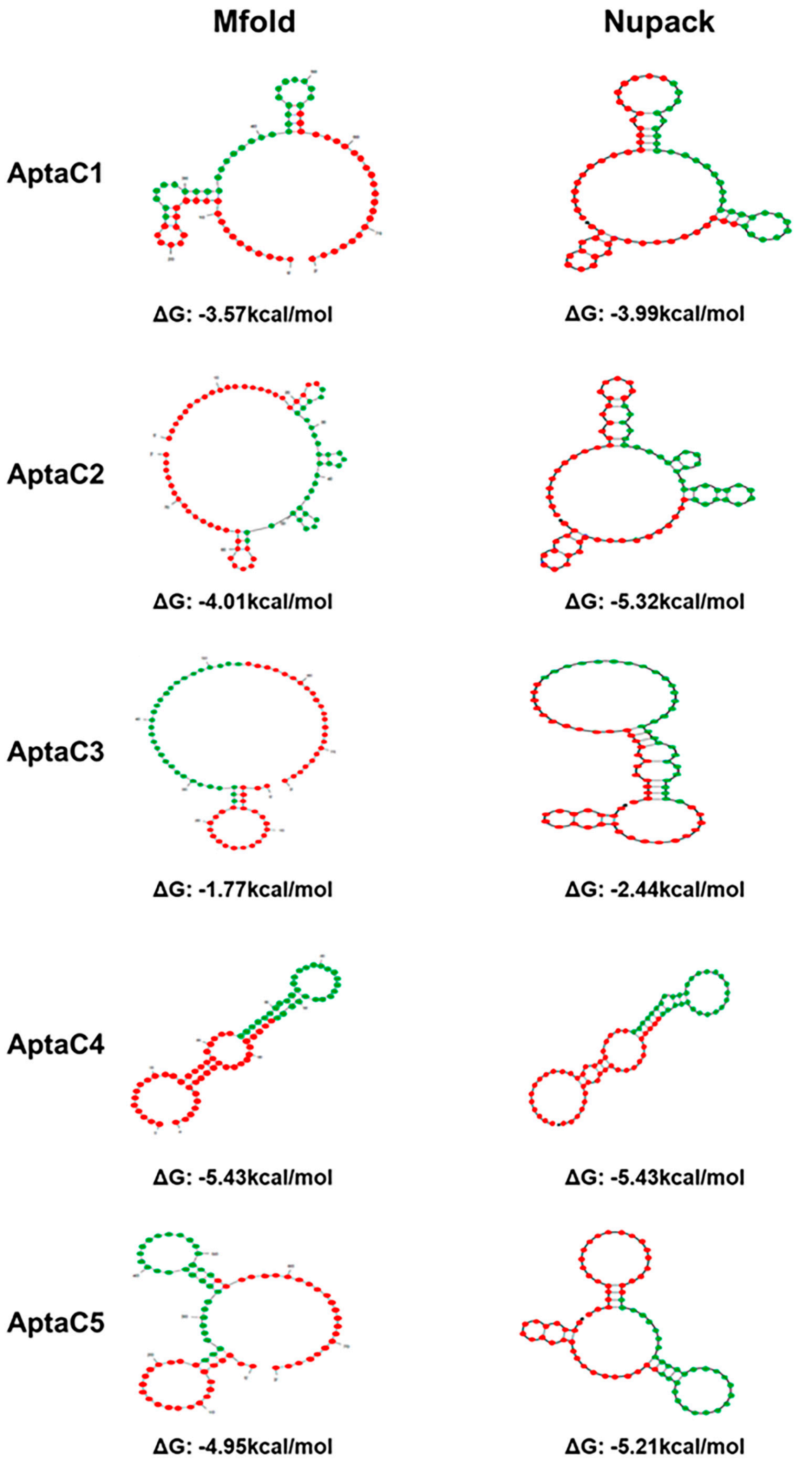

Figure S1: Characterization of secondary aptamer structures.

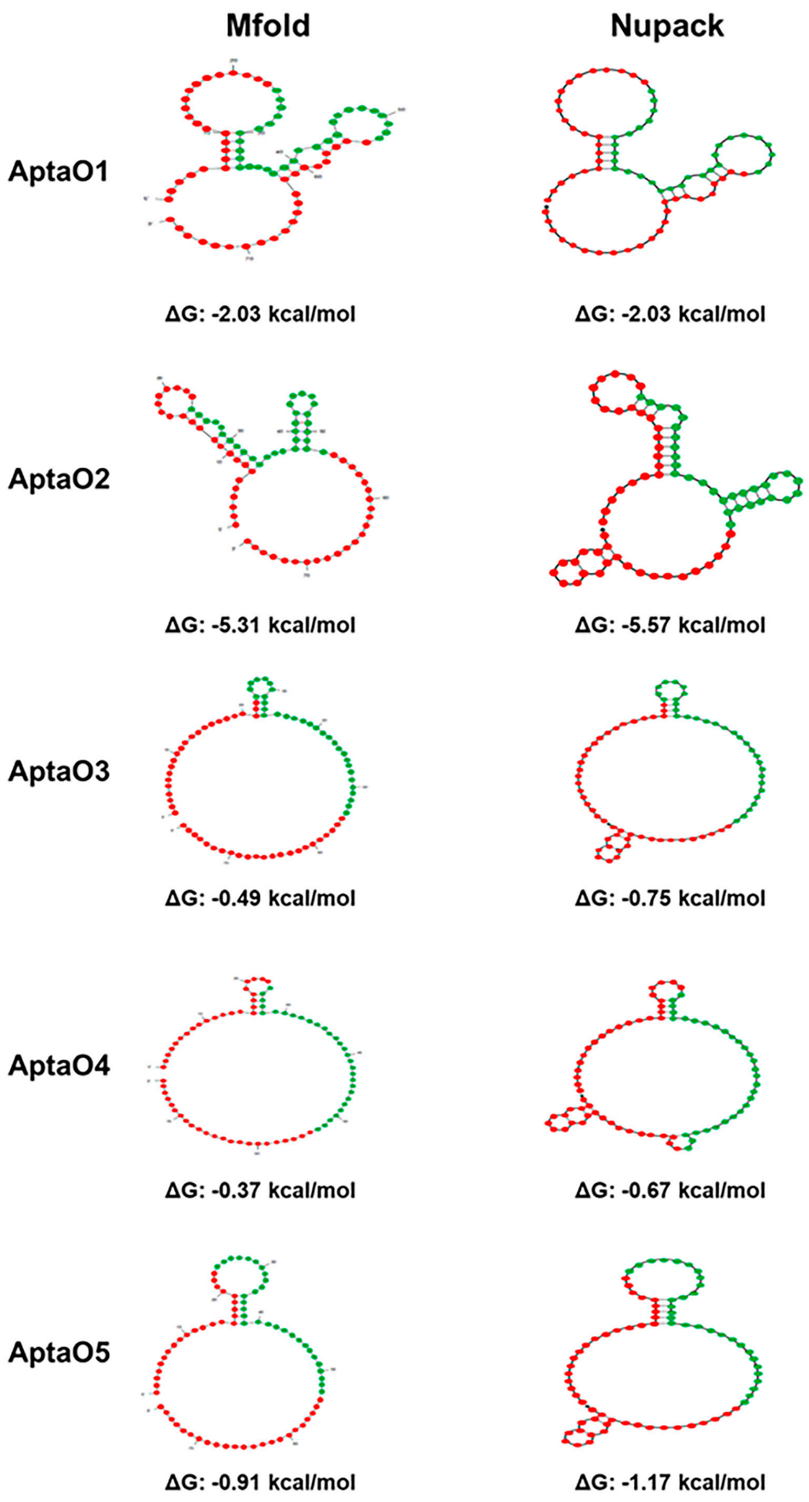

Figure S2: Characterization of secondary aptamer structures.

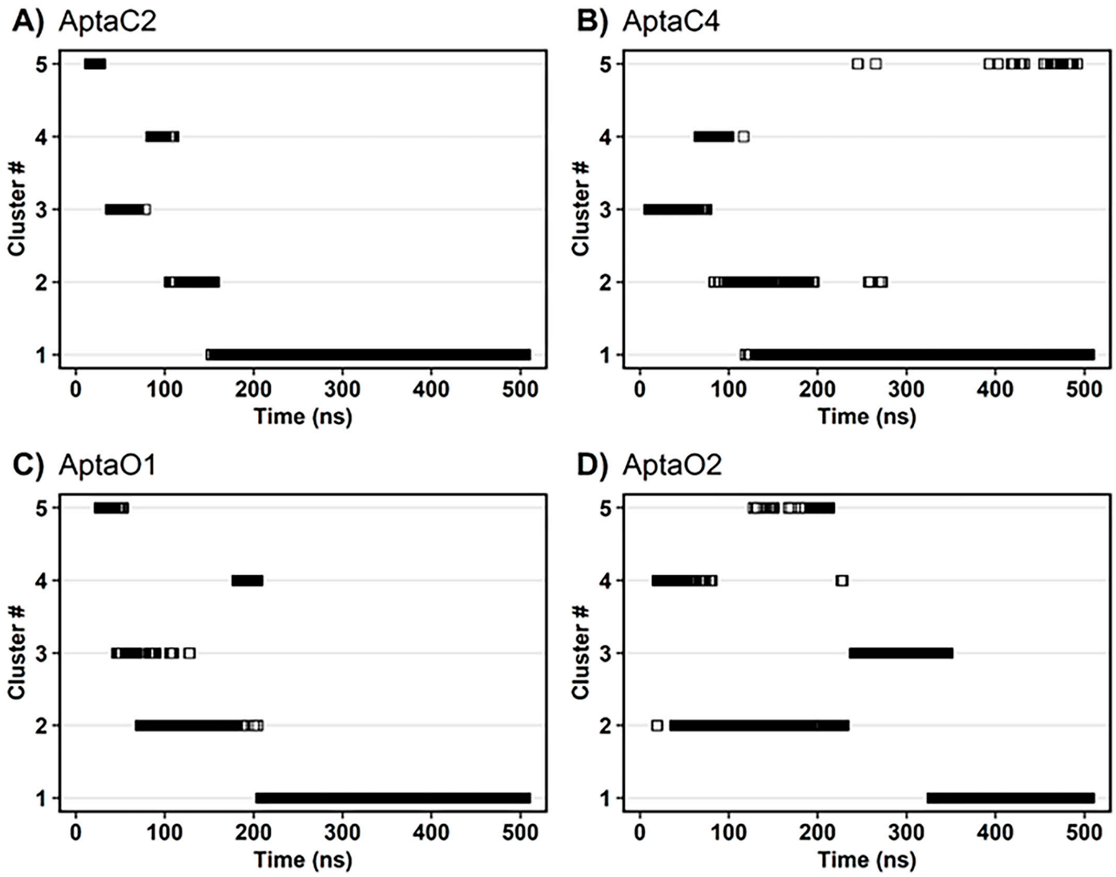

Figure S3: Root mean square clusters throughout the molecular simulations.
